# Improved Depiction of Pterygopalatine Fossa Anatomy Using Ultrahigh-Resolution Magnetic Resonance Imaging at 7 Tesla

**DOI:** 10.1100/2012/691095

**Published:** 2012-06-18

**Authors:** K. P. Q. Oomen, F. A. Pameijer, J. J. M. Zwanenburg, G. J. Hordijk, J. A. De Ru, R. L. A. W. Bleys

**Affiliations:** ^1^Department of Otolaryngology, UMC Utrecht, P.O. Box 85060, 3584 CG Utrecht, The Netherlands; ^2^Department of Radiology, UMC Utrecht, P.O. Box 85060, 3584 CG Utrecht, The Netherlands; ^3^Department of Radiotherapy, UMC Utrecht, P.O. Box 85060, 3584 CG Utrecht, The Netherlands; ^4^Department of Otolaryngology, Central Military Hospital, P.O. Box 90000, 3584 CX Utrecht, The Netherlands; ^5^Department of Anatomy, UMC Utrecht, P.O. Box 85060, 3584 CG Utrecht, The Netherlands

## Abstract

*Purpose*. To study the anatomy of the pterygopalatine fossa (PPF) using ultrahigh-resolution magnetic resonance imaging. *Methods*. A human cadaveric tissue block containing the pterygopalatine fossa was examined on a clinical 7-Tesla magnetic resonance imaging system. Subsequently, cryosections of the tissue block were created in a coronal plane. The cryosections were photographed and collected on adhesive tape. The on-tape sections were stained for Mallory-Cason, in order to detail the anatomic structures within the fossa. Magnetic resonance images were compared with surface photos of the tissue block and on-tape sections. *Results*. High-resolution magnetic resonance images demonstrated the common macroscopic structures in the PPF. Smaller structures, best viewed at the level of the operation microscope, which have previously been obscured on magnetic resonance imaging, could be depicted. Some of the orbital pterygopalatine ganglion branches and the pharyngeal nerve were clearly viewed. *Conclusions*. In our experience with one human cadaver specimen, magnetic resonance imaging at 7 Tesla seems effective in depicting pterygopalatine fossa anatomy and provides previously unseen details through its demonstration of the pharyngeal nerve and the orbital pterygopalatine ganglion branches. The true viability of depicting the pterygopalatine fossa with ultrahigh-resolution MR will depend on confirmation of our results in larger studies.

## 1. Introduction

The pterygopalatine fossa (PPF) is an inverted pyramidal space located inferior to the orbital apex, which contains the pterygopalatine ganglion (PPG) and various arteries, veins, lymphatics, and nerves. Preganglionic parasympathetic facial nerve fibres synapse in the PPG, while postganglionic sympathetic fibres from the superior cervical ganglion and sensory fibres from the maxillary nerve pass through the ganglion without synapsing. The PPF communicates with the orbit, nasal cavity, and oral cavity, and through the orbit with the maxillary sinus and upper teeth, which makes it an important cranial neurovascular crossroad as well as a common site for invasion and perineural spread of malignant disease [1]. The neural content of the PPF plays an important role in the pathophysiology of pain syndromes with cranial autonomic features such as cluster headache and Sluder's neuralgia [2, 3]. These syndromes are invalidating and may require invasive treatment, such as PPG blockage, for refractory cases [4]. Thus, studying the PPF in head and neck imaging is of importance, for both diagnostic and preoperative purposes.

 Previous studies have shown that on magnetic resonance imaging (MRI) at 1.5 Tesla, small PPF structures remain obscured, whereas the PPG and the sphenopalatine segment of the maxillary artery and some of its branches can easily be identified [5]. The recent development of MRI at 7 Tesla (7 T MRI) holds the promise of an increased signal-to-noise ratio (SNR). In various human anatomical regions, the increased SNR of 7 T MRI has been used to produce high-definition images with ultrahigh resolution and identification of previously unidentified detail [6, 7].

The aim of the present study is to correlate MR findings to cryosections in order to determine which part of the PPF and its contents can be identified on 7 T MRI.

## 2. Materials and Methods

### 2.1. Tissue Preparation

One undissected human head was obtained from a male postmortem, 73 years of age. The head was perfused with 0.9% NaCl under physiologic pressure and frozen at −25°C.

### 2.2. Whole-Mount Preparation

The head was transected on the median plane using a band saw and trimmed to a block containing the PPF and parts of the orbit, nasal and paranasal cavities, and oral cavity.

### 2.3. Imaging

The specimen was examined on a whole-body clinical 7 T MRI system (Philips Healthcare, Cleveland, OH, USA), using a transmit/receive head coil with a 16-channel receive coil (Nova Medical, Wilmington, MA, USA). During the MR examination, the specimen was submersed entirely in fomblin (Solvay Solexis, Bollate, Italy) to provide susceptibility matching, thereby contributing to accurate *B*_0_ shimming [8]. A 3D (volumetric) multiecho gradient echo sequence was applied with the following scan parameters: field of view (FOV) 100 × 81 × 60 mm^3^, acquisition matrix 332 × 270, 199 slices, slice thickness 0.3 mm, acquired resolution 0.3 × 0.3 × 0.3 mm^3^ (voxel volume 27 nL), TR 158 ms, TE 3.3 ms, fat suppression with SPAIR (inversion delay 50 ms), and bandwidth 427 Hz/pixel. The acquisition duration was approximately 5 hours and 32 minutes. Parameters were consistent with a T1 weighted scan.

### 2.4. Cryomicrotome Sectioning

After MR scanning, the whole-mount specimen was fixed in formaldehyde 4%, followed by rinsing in running tap water for several days. After overnight impregnation in 1% carboxymethylcellulose (CMC), the specimen was frozen and embedded in 1% CMC in the cryomicrotome (PMV 450MP; Palmstiernas Instruments AB, Stockholm, Sweden) at −25°C. The specimen was positioned in the cryomicrotome in an orientation matching its position on the MR images. Cryosections were created in a coronal plane, with a section thickness of 25 *μ*m.

Pictures of the tissue block surface in the region of the PPF were taken every 75 *μ*m of sectioning. Sections were collected on wide adhesive tape every 375 *μ*m of sectioning. The on-tape sections were stained with a modified Mallory-Cason procedure [9] and mounted on cardboard. A corresponding photograph and section were assigned to each of the MR images, taking into account the slice thickness, slice gap, and slice interval.

## 3. Results

**Figure 1:**
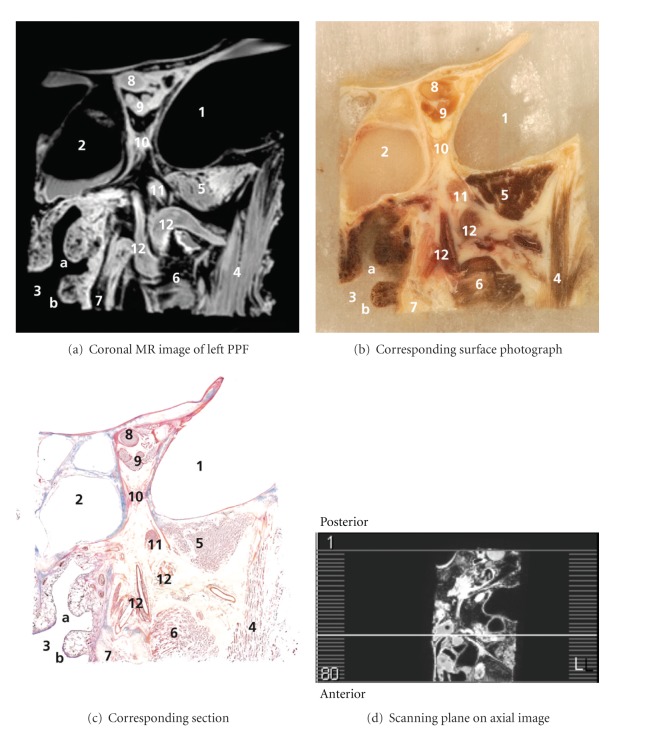
1: middle cranial fossa, 2: sphenoidal sinus, 3: nasal cavity with (a) middle concha and (b) inferior concha, 4: temporal muscle, 5: lateral pterygoid muscle, superior head, 6: lateral pterygoid muscle, inferior head, 7: medial pterygoid muscle, 8: optic nerve, 9: common annular tendon (of Zinn) with origins of the medial, inferior, and lateral rectus muscles, 10: orbital or Muller's muscle, 11: maxillary nerve, and 12: sphenopalatine artery.

**Figure 2:**
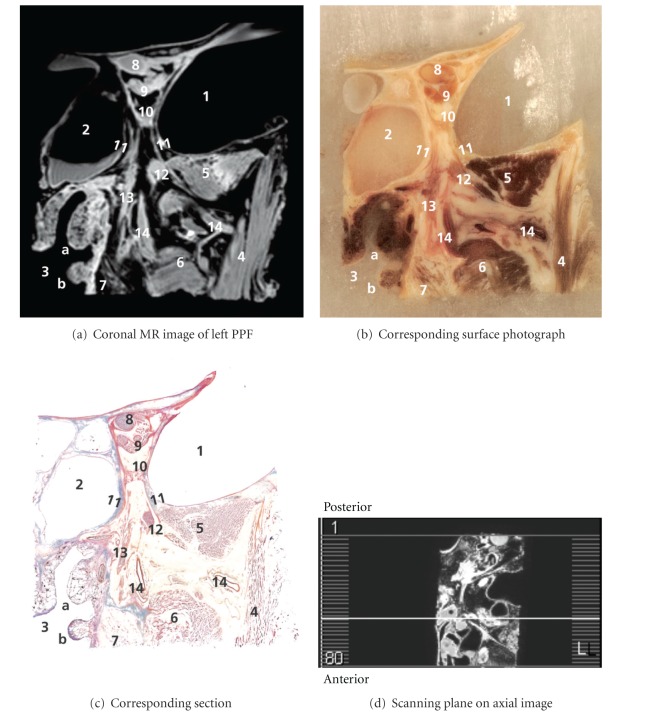
1–10: see Figure 1. 11: orbital PPG branches, 12: maxillary nerve, 13: greater palatine nerve, and 14: maxillary artery continuing as sphenopalatine artery.

**Figure 3:**
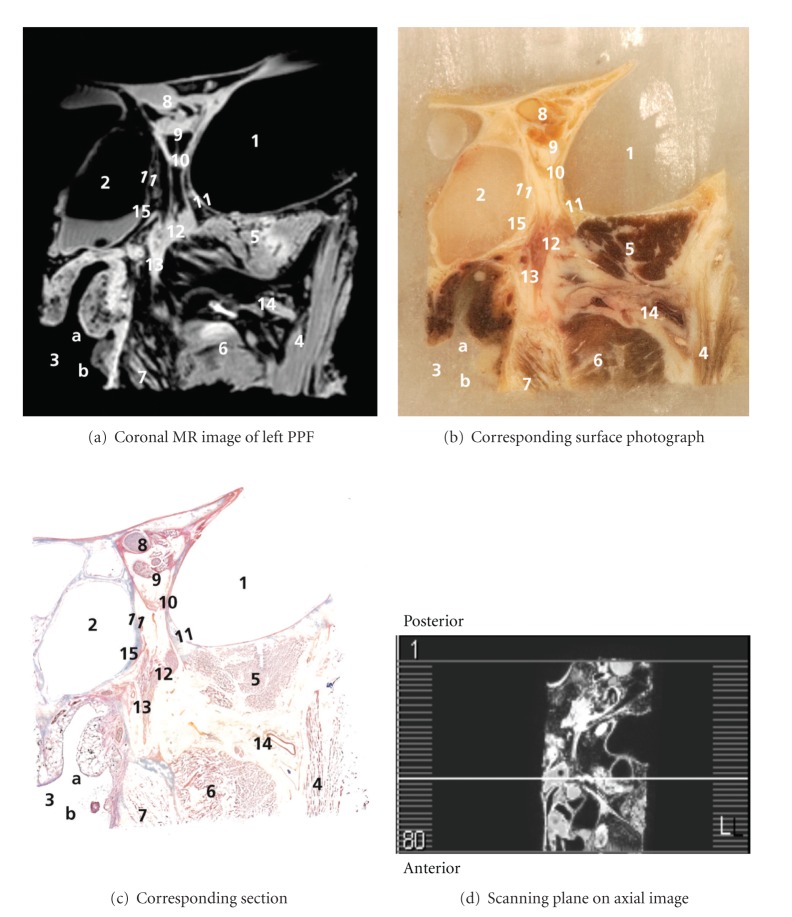
1–11: see Figure 2. 12: PPG, 13: greater palatine nerve, 14: maxillary artery, and 15: pharyngeal nerve.

**Figure 4:**
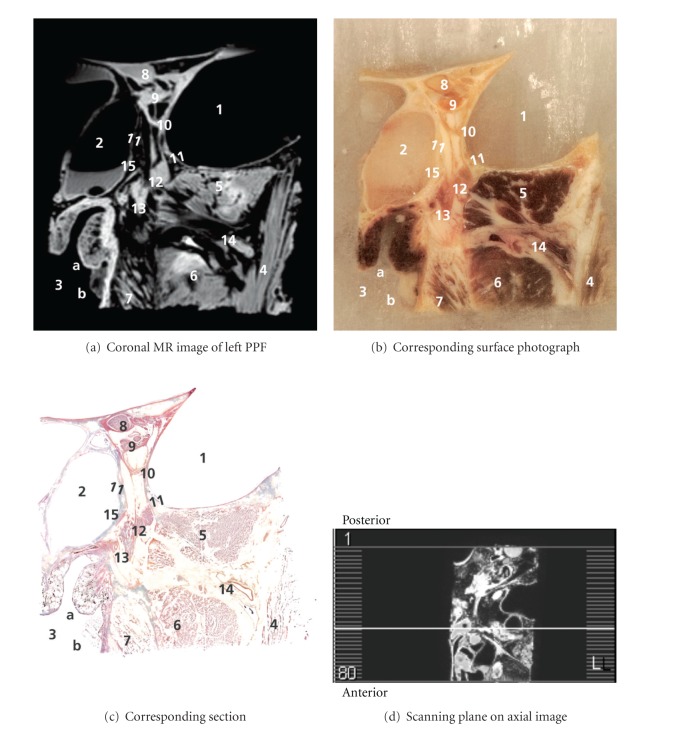
1–12: see Figure 3. 13: origin of greater palatine nerve, 14: maxillary artery, and 15: pharyngeal nerve.

**Figure 5:**
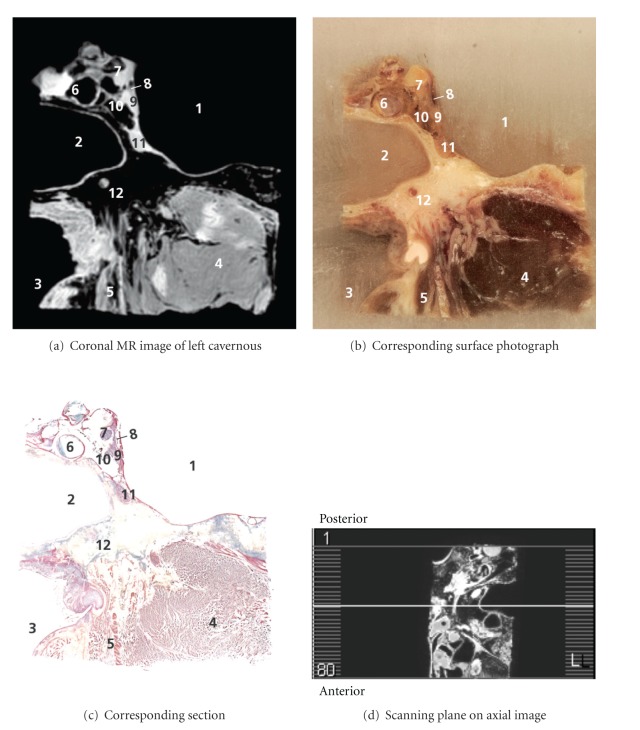
1–3: see Figure 4. 4: lateral pterygoid muscle, 5: tensor veli palatini muscle, 6: internal carotid artery, 7: oculomotor nerve, 8: trochlear nerve, 9: ophthalmic nerve, 10: abducens nerve, 11: maxillary nerve, and 12: nerve of the pterygoid canal or Vidian nerve.

Correlations of anatomical findings and coronal MR images are shown in an anteroposterior series of Figures 1 to 5. Figures 1 to 4 show several common anatomical structures in and around the PPF. The sphenoidal sinus (SS), middle cranial fossa, and nasal cavity were used as orientation points. In the lateral nasal wall, the middle and inferior conchas were found. Lateral to the PPF, several structures were found with intermediate signal intensity on MR and an orange red Mallory-Cason stain, consistent with muscle tissue. From medial to lateral, the medial pterygoid muscle (MPM), lateral pterygoid muscle (LPM) with its superior and inferior heads, and temporal muscle were identified.

The optic nerve (ON) was identified from its origin in the optic chiasm to its position just lateral to the SS. Caudal to the ON, the common annular tendon (of Zinn) was identified, a ring of fibrous tissue surrounding the ON at its entrance at the apex of the orbit, which forms the origin of the four straight extraocular muscles. The common origin of the medial, inferior, and lateral rectus muscle was clearly depicted.

Caudal to the common annular tendon, a structure was found with high signal intensity on MR and an orange red Mallory-Cason stain on on-tape cryosections, consistent with muscle tissue. This structure could be identified as the orbital or Muller's muscle (MM), which consists of smooth muscle overlying the inferior orbital fissure (IOF). In the PPF, the maxillary nerve (V2) was found and followed along its course and mergence with the PPG. A detailed view of the PPG is included in Figures 3 and 4. Even several small nerve branches of the PPG were visualized. From the PPG, the greater palatine nerve was found running caudally, and three slender structures were clearly viewed running in a cranial direction into the IOF, with high signal intensity on MR and a light red Mallory-Cason stain consistent with neural tissue. These neural structures could be identified as orbital PPG branches and had connections to MM. One of these orbital branches, however, seemed to stem from the distal part of V2, as is shown in Figures 2 through 4. Figures 3 and 4 reveal a structure that originated from the PPG and ran in a medial direction. This structure showed high signal intensity on MR and a light red Mallory-Cason stain, consistent with neural tissue. This neural structure was identified as the pharyngeal nerve, in its course towards the palatovaginal canal. From a lateral direction, a remarkably tortuous structure, with a low intensity on MR and a dark red Mallory-Cason stain with a distinct lumen, consistent with vascular tissue, was found running between the superior and inferior head of the LPM, toward and into the PPF (Figures 1 and 2). This vascular structure was identified as the maxillary artery continuing as the sphenopalatine artery (SPA).

In the most posterior image, Figure 5, the cavernous sinus and the structures related to its medial wall were demonstrated. The internal carotid artery, the oculomotor nerve, the trochlear nerve, the abducens nerve, the ophthalmic nerve (V1), and V2 were all clearly visible. The nerve of the pterygoid canal or Vidian nerve was found traversing the base of the pterygoid process in the floor of the SS.

## 4. Discussion

Our study demonstrates that ex vivo MR imaging of the PPF at 7 T provides excellent depiction of PPF content, specifically the PPG and some of its branches. Some of the orbital branches and the pharyngeal nerve were clearly visible, and the orbital branches could be followed toward one of their targets, MM.

Comparison of our findings with those in previous radiological studies is hampered by the fact that few studies are available on MRI appearance of the PPF. Following the introduction of high-resolution computed tomography (CT), several investigators have compared CT findings of normal and pathological anatomy of the PPF with findings in cadaver specimens [10, 11]. As expected, previous CT studies of the PPF have focused on its boundaries and communications, rather than its content [12, 13]. The few MR studies that are available focus on perineural tumor spread in the PPF, which excludes a detailed search for structures such as the PPG and its communications [5, 14–16]. Although the palatovaginal canals are commonly depicted on MR [11], a clear depiction of the pharyngeal nerve is hitherto undescribed in radiological studies of the PPF. Rumboldt et al. [11] have described structures in the palatovaginal canal that presumably correspond to the pterygovaginal artery and, possibly, the pharyngeal nerve. However, their findings were only visible as flow voids or low-signal-intensity structures on T1-weighted images, and the presumed structures were not visible in great detail. The orbital PPG branches have never been described in radiological PPF studies. Some of our findings correspond to those in previous anatomical studies. A previous endoscopic study of the anatomical relations of the PPG revealed a remarkably tortuous portion of the SPA along its course in the PPF, suggestive of a potentiality of vascular compression of the PPG as a causative factor in headaches with ipsilateral cranial autonomic features [17]. Our images of the SPA are in line with this description, although direct compression of the SPA on the PPG was not present. Ruskell (1970) described the orbital PPG branches in primates and humans in detail [18] and demonstrated that orbital branches originate in the PPG and reach the orbit by passing through the IOF. According to Ruskell, the orbital branches penetrate the orbital smooth muscle (MM) and pass adjacent to the periosteum of the orbit at the apex either medially or laterally. The orbital PPG branches depicted on 7 T MRI in our study demonstrated the same configuration. A recent cadaver study on neurochemical characterization of PPG branches in humans demonstrated a group of orbital branches that stem from the distal part of the maxillary or infraorbital nerve, the anterior group of orbital PPG branches [19]. These findings were confirmed in our study. In a recent human cadaver study by Oomen et al. [20], macro- and microdissection of whole-mount preparations of the PPF combined with nerve-specific staining demonstrated a previously undescribed orbital PPG branch, which runs between the PPG and the V1. This specific orbital PPG branch could not be demonstrated in our MR study.

The improved depiction of the neural PPG connections, such as the orbital branches and the pharyngeal nerve, could become clinically important once the pathophysiology of facial pain is completely understood, including the exact pain pathways. In treatment of facial pain, these insights might hold the promise of development of selective nerve blocks in this area, in which ultrahigh-resolution imaging of the PPF at 7 T could be of aid as a preoperative measure.

Although our results seem promising, the fact that this concerns a cadaver study and not an in vivo study has to be taken into account. The current acquisition duration of the scan was 5 hours and 32 minutes, which for obvious reasons is not applicable to living subjects. Furthermore, cadaver images differ in signal intensities from in vivo images, which could account for subtle changes in the appearance of anatomical structures. The true viability of depicting the PPF with ultrahigh-resolution MR, therefore, depends on confirmation of our positive results in larger studies with living human subjects.

In conclusion, in our experience with one human cadaver specimen, MR of the PPF at 7 T provides excellent depiction of PPF content and demonstrates hitherto radiologically obscured anatomical details such as the orbital PPG branches and the pharyngeal nerve. High-resolution MR at 7 T could potentially contribute to an improved diagnostic and preoperative evaluation of the PPF and its content.

##  Conflict of Interests

The authors declare that they have no conflict of interests.
